# Sertaconazole-repurposed nanoplatform enhances lung cancer therapy via CD44-targeted drug delivery

**DOI:** 10.1186/s13046-023-02766-2

**Published:** 2023-07-29

**Authors:** Ruolan Liu, Qiong Li, Siyuan Qin, Ling Qiao, Mei Yang, Shanshan Liu, Edouard C. Nice, Wei Zhang, Canhua Huang, Shaojiang Zheng, Wei Gao

**Affiliations:** 1 School of Basic Medical Sciences, Chengdu University of Traditional Chinese Medicine, Chengdu, 611137 China; 2 State Key Laboratory of Biotherapy and Cancer Center, West China Hospital, and West China School of Basic Medical Sciences and Forensic Medicine, Sichuan University, and Collaborative Innovation Center for Biotherapy, Chengdu, 610041 China; 3 School of Pharmacy, Zunyi Medical University, Zunyi, 563000 China; 4 Department of Biochemistry and Molecular Biology, Monash University, Clayton, VIC 3800 Australia; 5 West China Biomedical Big Data Center, West China Hospital, Sichuan University, Chengdu, 610041 China; 6 Mental Health Center and Psychiatric Laboratory, the State Key Laboratory of Biotherapy, West China Hospital of Sichuan University, Chengdu, China; 7 Hainan Cancer Center of The First Affiliated Hospital, Key Laboratory of Tropical Cardiovascular Diseases Research of Hainan Province, Hainan Women and Children’s Medical Center, Hainan Medical University, Haikou, 571199 China; 8 Clinical Genetics Laboratory, Affiliated Hospital & Clinical Medical College of Chengdu University, Chengdu, 610081 China

**Keywords:** Sertaconazole, Drug repurposing, Lung cancer, Nanoparticle, Targeted delivery

## Abstract

**Background:**

Lung cancer is one of the most frequent causes of cancer-related deaths worldwide. Drug repurposing and nano-drug delivery systems are attracting considerable attention for improving anti-cancer therapy. Sertaconazole (STZ), an antifungal agent, has been reported to exhibit cytotoxicity against both normal and tumor cells, and its medical use is limited by its poor solubility. In order to overcome such shortcomings, we prepared a drug-repurposed nanoplatform to enhance the anti-tumor efficiency.

**Methods:**

Nanoplatform was prepared by thin film dispersion. Drug release studies and uptake studies were measured in vitro. Subsequently, we verified the tumor inhibition mechanisms of HTS NPs through apoptosis assay, immunoblotting and reactive oxygen species (ROS) detection analyses. Antitumor activity was evaluated on an established xenograft lung cancer model in vivo.

**Results:**

Our nanoplatform improved the solubility of sertaconazole and increased its accumulation in tumor cells. Mechanistically, HTS NPs was dependent on ROS-mediated apoptosis and pro-apoptotic autophagy to achieve their excellent anti-tumor effects. Furthermore, HTS NPs also showed strong inhibitory ability in nude mouse xenograft models without significant side effects.

**Conclusions:**

Our results suggest that sertaconazole-repurposed nanoplatform provides an effective strategy for lung cancer treatment.

**Supplementary Information:**

The online version contains supplementary material available at 10.1186/s13046-023-02766-2.

## Introduction

Lung cancer is a malignant tumor originating from the cells lining the bronchi and parts of the lung such as the bronchioles or alveoli. At present, it is the most commonly diagnosed cancer (accounting for 11.6%), and accounts for 18.4% of total cancer deaths [1]. With societal and economic developments, the incidence and mortality of lung cancer continue to rise, seriously endangering world health [2]. At present, the first choice of treatment for lung cancer is surgical resection combined with postoperative adjuvant chemotherapy, which is effective for early-stage lung cancer. However, there is still a lack of effective treatment for patients with advanced lung cancer [3]. Therefore, it is important to actively explore novel treatment options for lung cancer to improve both the survival and quality of life of lung cancer patients. Considering the high cost (> 2 billion), long time (10–15 years), and low success rate of traditional drug development [4, 5], drug repurposing provides an advantage in substantially simplifying the process from drug discovery to approval for clinical applications [6]. Drug repurposing is an emerging approach to identifying new indications for existing drugs or overcoming their physical limitations such as insolubility, and has already been applied in the clinic to improve therapeutic efficacy [7].

Sertaconazole is an imidazole antifungal agent with significant antifungal activity against pathogenic fungi, including yeast-like fungi, skin fungi, and other filamentous fungi [8]. Several studies have revealed the beneficial pharmacological activity of sertaconazole in various types of cancer, indicating potential anti-tumor effects [9, 10]. Moreover, sertaconazole can stabilize the expression of tumor necrosis factor receptor type 1 related death domain protein (TRADD) through the ubiquitination degradation pathway, and up-regulate its content, eventually leading to the dephosphorylation of threonine protein kinases, and triggering apoptosis of lung cancer cells [11]. However, the limited water-solubility and toxicity to normal cells hinder its practical application [9, 12]. Recently, the use of nanocarriers to deliver drugs has become a really attractive solution [13]. In particular, numerous amphiphilic polymeric micelles, like multifunctional nanocarriers, have been proven to be effective in encapsulating various small molecular therapeutic drugs and delivering them to tumor tissues [14, 15].

CD44, a multistructural and multifunctional cell surface glycoprotein, plays an essential role in physiological activities in normal cells and pathological activities in cancer cells, such as cell proliferation, differentiation, migration, angiogenesis, presentation of cytokines, chemokines, and growth factors to the corresponding receptors, and docking of proteases at the cell membrane, as well as in signaling for cell survival [16]. The expression of CD44 is higher in multiple tumor cells than that in corresponding normal tissues [17], such as lung cancer [18]. As a natural macromolecular polymer, hyaluronic acid (HA) is often used for targeted therapy of tumors due to its specific binding to high-expression CD44 receptor on tumor cell membranes [19, 20]. D-α-tocophenol-polyethylene glycol 1000 succinate (TPGS) is a water-soluble derivative of natural vitamin E with an amphiphilic structure consisting of a lipophilic alkyl tail and a hydrophilic polar head. The solubilization of TPGS has been widely used in soluble drug delivery systems. More studies have shown that the poorly soluble drug formononetin incorporated into phospholipid/TPGS micelles, the solubility of formononetin was improved and its inhibitory effect on lung cancer was enhanced [21]. In another study, resveratrol loaded TPGS liposome drug-delivery System for brain targeting. The study demonstrated superior cellular uptake of TPGS-modified liposomes in C6 glioma cancer cell lines and higher plasma half-life of TPGS-resveratrol liposomes compared to resveratrol in uncoated liposomes [22]. In a recent study, HA and TPGS functionalized AuMSS nanorods were used to construct a tumor-targeted photothermal nanomedicine for the first time, which can significantly induce the death of HeLa cancer cells [23]. In another study, the authors prepared a self-assembled HA-SMA-TPGS encapsulated with a poorly water-soluble potent curcumin analogue (CDF) to form nanomicelles for targeting TNBC. HA-SMA-TPGS-CDF has shown excellent nanoparticle properties for parenteral delivery. It was demonstrated that HA-SMA-TPGS-CDF had a higher cell killing effect compared to untargeted SMA-TPGS micelles [24]. Overall, there have been numerous examples of the application of the delivery system based on hyaluronic acid and TPGS in various cancer therapies, suggesting that the potential use of this system for other anticancer drugs against other types of cancer.

**Scheme 1:**
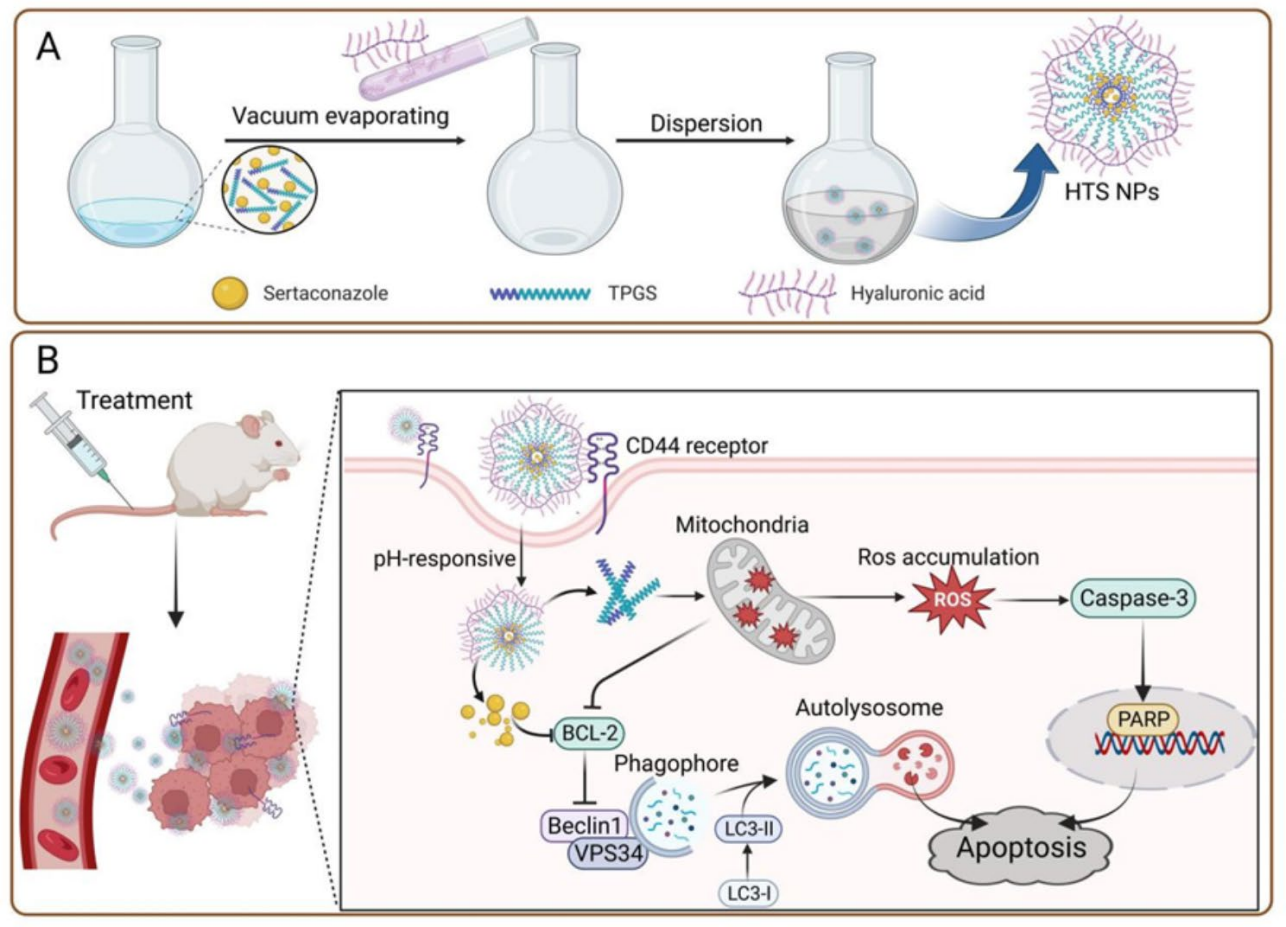
(**A**) Schematic illustration showing the preparation of HTS NPs and (**B**) the mechanism of HTS NPs for lung cancer therapy

Herein, we designed a nanoplatform HA-TPGS-STZ NPs (defined as HTS NPs) for repurposing antifungal drugs (Scheme 1). In this nanoplatform, HA conferred site-specific targeting and pH-responsive delivery behavior to enhance intracellular aggregation and STZ release. In addition, TPGS improved the solubility of STZ by solubilization. Mechanistically, STZ can effectively induce the intact autophagic flow, which contributes to the occurrence of apoptosis, while TPGS can produce a large amount of ROS to induce mitochondria-related apoptosis and synergistically induce cell death, with negligible adverse effects on normal tissues (Scheme 1B). Therefore, our HTS NPs nanoplatform have a significant synergistic effect and provide a promising therapeutic strategy for the treatment of lung cancer.

## Materials and methods

### Materials

Sertaconazole nitrate (HY-B0736A) was obtained from MedChemexpress Biotechnology Inc. (American). Hyaluronic acid (MW = 36-1400 kDa) was purchased from Shanghai Aladdin Biotech Co., Ltd.Chlorin e6 (Ce6) was purchased from Glpbio Technology Inc. (Montclair, California). TPGS, Dimethylsulfoxide (DMSO), N-acetyl cysteine (NAC), streptomycin, penicillin and crystal violet were bought from Millipore Sigma. 3-(4,5-dimethyl-2-thiazoyl)-2,5-diphenyltetra zolium bromide (MTT) were obtained from Sigma-Aldrich Co., Ltd. Dulbecco’s modified Eagle’s medium (DMEM) and fetal bovine serum (FBS) was obtained from ThermoFisher Biochemical Products (Beijing) Co., Ltd. ROS assay kit (Catalog no. s0033s), LDH (Catalog no. C0016), Total Superoxide Dismutase Assay kit (Catalog no. S0101S), Total Antioxidant Capacity Assay kit (Catalog no. S0119), Lipid Peroxidation MDA Assay kit (Catalog no. S0131S), Calcein-AM/PI Cytotoxicity Assay kit (Catalog no. C2015S), Dihydroethidium (Catalog no. S0063) were provided by Beyotime Biotechnology. 5-ethynyl-2’-deoxyuridine (EdU) labeling assay kit (Catalog no. 40275ES6) and Annexin V-FITC/PI Apoptosis Detection kit (Catalog no. 40302ES60) was purchased from Yeasen Biotech (Shanghai) Co., Ltd. 2′,7′-Dichlorofluorescein diacetate (DCFH-DA) was purchased from Sigma-Aldrich. Transmission electron microscopy (TEM) images were obtained by a transmission electron microscope (HITACHI HT7800, Japan). The UV-Vis spectrum was obtained using a UV-Vis spectrometer (UV2700, Shimadzu, Japan). The levels of LDH, and cell viability were measured using a microplate reader (Synergy H1, BioTek, USA). The fluorescence images were obtained from a fluorescent imaging analysis (Olympus, Tokyo, Japan). Blood biochemistry analysis was conducted by an Auto biochemical analyzer (Chemray 240, Rayto, China).

### The preparation of HTS NPs

HTS NPs was prepared according to the thin film hydration method (Scheme 1 A). Briefly, Sertaconazole (2.5 mg) and TPGS (10 mg) were dissolved in 5 mL methanol. The mixture was evaporated via a rotary evaporator at 37 °C under reduced pressure to remove the methanol and form a thin lipid film. The resultant dry film was hydrated with the 5 mL HA solution (2 mg/mL). These suspensions were subsequently treated with an ultrasonic cell disruptor for 3 min (duration 5 s, interval 5 s) to prepare HTS NPs. For bioluminescent imaging, Chlorin e6-loaded nanoparticles (HT-Ce6 NPs) were prepared in the same way, except sertaconazole (2.5 mg) was used instead of Ce6 (3 mg). All prepared nanoparticles were stored in the refrigerator at 4 °C.

### Characterization of HTS NPs

Hydrodynamic diameter and zeta potential of composite nanoparticle were determined by dynamic light scattering (DLS) of Malvern Zetasizer (ZSE). The appearance was visualized by transmission electron microscope (TEM). The release of STZ from HTS NPs was carried out using a dialysis method. Briefly, HTS NPs were placed into dialysis bags, each dialysis bag containing 1.5 mL solution. After sealing and immerged in 40 mL PBS (pH 5.0 and 7.4) at 37 °C with rotation at 80 rpm. 1 mL of release medium was then withdrawn and replaced with 1 mL fresh medium at predetermined times. The amount of STZ was determined by UV-vis absorption spectra according to a standard curve. All assays were conducted in parallel in triplicate.

### Cell culture

Human non-small cell lung cancer cell lines H460, A549 and human normal bronchial epithelial cells 16HBE were purchased from the ATCC. The cells were maintained in high glucose Dulbecco’s modified Eagle Medium (DMEM, Gibco) supplemented with 10% FBS (Biowest), and 1% penicillin-streptomycin (Hyclone). All cells were grown at 37 °C and 5% CO_2_ in a humidified incubator.

### In vitro cellular uptake

To investigate the cellular uptake, 16HBE, A549 and H460 cells were seeded in 6-well culture plates at a density of 2 × 10^5^ cells per well for 24 h at 37 °C. Culture medium containing free Ce6 and HT-Ce6 NPs. The final Ce6 concentration in each well was 20 µM. After incubation for 1, 2, 4, and 6 h, cells were washed with PBS three times, harvested and resuspended in 500 µL PBS. The cellular uptake was quantified using flow cytometry (FC) as mentioned above.

### Cell viability assay

Cell viability was assessed using the MTT assay. 16HBE, A549 and H460 cells were seeded into 96-well plates (3000 cells/well) for 24 h at 37 °C. After cell adherence, cells were treated with different concentrations of TPGS, STZ, TS NPs and HTS NPs for 4 h. Then, 10 µL of 5 mg/mL MTT dissolved in PBS was added to each well and incubated for 2 h in 37 °C. The medium was aspirated, and 100 µL of DMSO was added to each well, the absorbance was measured at 570 nm using a UV-vis spectrophotometer. For colony formation assay, H460 and A549 cells were incubated in 24-well plates (1 × 10^3^ cells/well) and incubated overnight. The cells were continuously cultured in the presence or absence various concentrations of the preparations. The clones were fixed with 4% paraformaldehyde for 30 min and stained with crystal violet. The clone formation rate was obtained by calculating the ratio of the crystal violet staining areas of the control and experimental groups.

### EdU proliferation assay

To measure cell proliferation, EdU proliferation assay was performed. Cells were plated in 96-well plates at a density of 5 × 10^4^ cells/well and incubated overnight. The next day, the cells were treated with TPGS, STZ, TS NPs and HTS NPs at various concentrations for 4 h at 37 °C. Then, the cells were stained with the EdU labeling assay kit (C10310-1) following the manufacturer’s recommendations. Cells were treated with 10 µM EdU and fixed with 4% paraformaldehyde. The sections were imaged using a fluorescence microscope.

### LDH release assay

H460 and A549 cells were placed in 96-well plates (3000 cells/well) and treated with TPGS, STZ, TS NPs and HTS NPs at various concentrations for 4 h at 37 °C. Then, the supernatant was transferred to the new 96-well plates for LDH analysis according to the supplier’s instruction.

### Apoptosis assay

H460/A549 cells were plated on 6-well plates at a density of 1 × 10^5^ cells/well, after grown for 24 h, the medium was removed and the cells were incubated with 10 µM of TPGS, STZ, TS NPs and HTS NPs for 24 h. Cells were then digested with trypsin and collected using pancreatin with no EDTA. The cells were rinsed twice with PBS, resuspended in 100 µL binding buffer, then mixed with 5 µL Annexin-V and 10 µL PI. The cells were incubated in the dark for 15 min and cells were analyzed using flow cytometry.

### Intracellular ROS generation assay

Intracellular ROS generation was assessed using the 2,7-dichlorodihydrofluorescein diacetate (DCFH-DA) kit. According to the manufacturer’s instruction, H460/A549 cells were seeded in plates 6-well plates (2 × 10^5^ cells/well) and incubated overnight. Then cells were subjected to different treatment. After incubating with 1.0 µM DCFH-DA at 37 °C for 20 min. Fluorescence intensity was evaluated by fluorescence microscopy and flow cytometry.

### Immunoblotting

Cells were harvested and washed with pre-cool PBS and then lysed with RIPA buffer (1% deoxycholate, 1% Triton X-100, 0.1% SDS supplemented with phosphatase inhibitor and protease inhibitor) and sonication for 30 s. Total lysates were subjected to SDS-PAGE, followed by transfer onto PVDF membranes, which were blocked with skimmed milk for 2 h at room temperature. After incubation with the indicated primary antibodies at 4 °C, the secondary antibodies were applied for another 2 h. The expression of proteins was detected via the ChemiScope 6000 Touch (Clinx, Shanghai) after incubation with Immobilon Western HRP Substrate (Millipore, WBKLS0050).

### Hemolysis test

The whole blood of mice was obtained by collecting blood through the eye socket, stirring with a glass plate, removing the fibrin, adding 10 times the volume of PBS, 10,000 g, centrifugation for 5 min, and carefully sucking the supernatant and discarding. The red cells were washed repeatedly until the supernatant was colorless and transparent. The obtained red blood cells were resuspended in sufficient PBS and prepared as a 4% (v/v) red blood cell suspension. STZ, HTS NPs mother liquor was subsequently diluted with PBS to a series of different concentrations. 0.5 mL distilled water and 0.5 mL red blood cell suspension were mixed and used as a positive control group. 0.5 mL PBS and 0.5 mL red blood cell suspension were mixed and used as negative control group. 0.5 mL red blood cell suspension was added into 0.5 mL sample with different concentrations, so that the final sample concentrations were STZ: 100 mg/mL, HTS NPs: 25 mg/mL, 50 mg/mL, 100 mg/mL. The above samples were incubated at 37 °C for 2 h and centrifuged at 10,000 g for 5 min. The samples were photographed, and the supernatant was collected. The absorbance at a wavelength of 570 nm was measured by a microplate reader. The hemolysis rate was calculated according to the following formula: Hemolysis rate (%) = (As-An)/(Ap-An) ×100%. As: the absorbance of the sample, An: the absorbance of the negative control, and Ap: the absorbance of the positive control.

### Animal subject

Male Balb/c mice (5 weeks old) were purchased from YaoKang Biological Technology Co., Ltd. (Chengdu). All animal experiments were carried out according to the ARRIVE (Animal Research: Reporting of In Vivo Experiments) guidelines. Furthermore, all animal studies were approved by the Institutional Animal Care and Use Committee of Sichuan University and the guidance of the National Research Council’s Guide for the Care and Use of Laboratory Animals.

### In vivo anti-tumor activity study

The H460 xenograft tumor model was established by subcutaneous injection of 100 µL H460 cells (1 × 10^6^ cells) into the back of nude mice. When the tumor volume reached 80–100 mm^3^, the mice were randomly divided into four groups (5 mice/group) and injected with STZ, TS NPs and HTS NPs (2 mg/kg of equivalent amount of STZ), whereas the control mice received saline via the tail vein every other day for a total of seven times. The tumor volume and body weight were measured every two days. The tumors were measured with a Vernier caliper and the volumes were calculated using the following equation: tumor volume (mm^3^) = (length×width^2^)/2. The stripped tumors from the sacrificed mouse in different groups were also weighed to evaluate the antitumor effect. The tumor and main organs of the mice were sectioned into thin slices for H&E and IHC staining. Images of the tumors were captured at the end of the study. Body weight changes were monitored in order to evaluate the systemic toxicity of the drugs and NPs.

### Living imaging

To examine the targeting properties of NPs, 2 mg HT-Ce6 NPs were intravenously injected in mice with the equal mass of T-Ce6 NPs (2 mg) and Ce6 (2 mg) used as controls. Mice were anaesthetized using 3% isoflurane for induction and 1.5% isoflurane for maintenance and photographed at 2, 4, 6, 8, 12, 24 h post-injection using the IVIS Lumina III system.

### Statistical analysis

Statistical analysis was performed using GraphPad Prism 8.0 software. Two-tailed Student t test was used to analyze statistical differences. Pearson correlation and linear regression were used to determine the concordance. Data were shown as means ± S.D. and significance were described as follows: *P < 0.05, **P < 0.01; ***P < 0.001.

## Results and discussion

### Preparation and characterization of HTS NPs

**Fig. 1:**
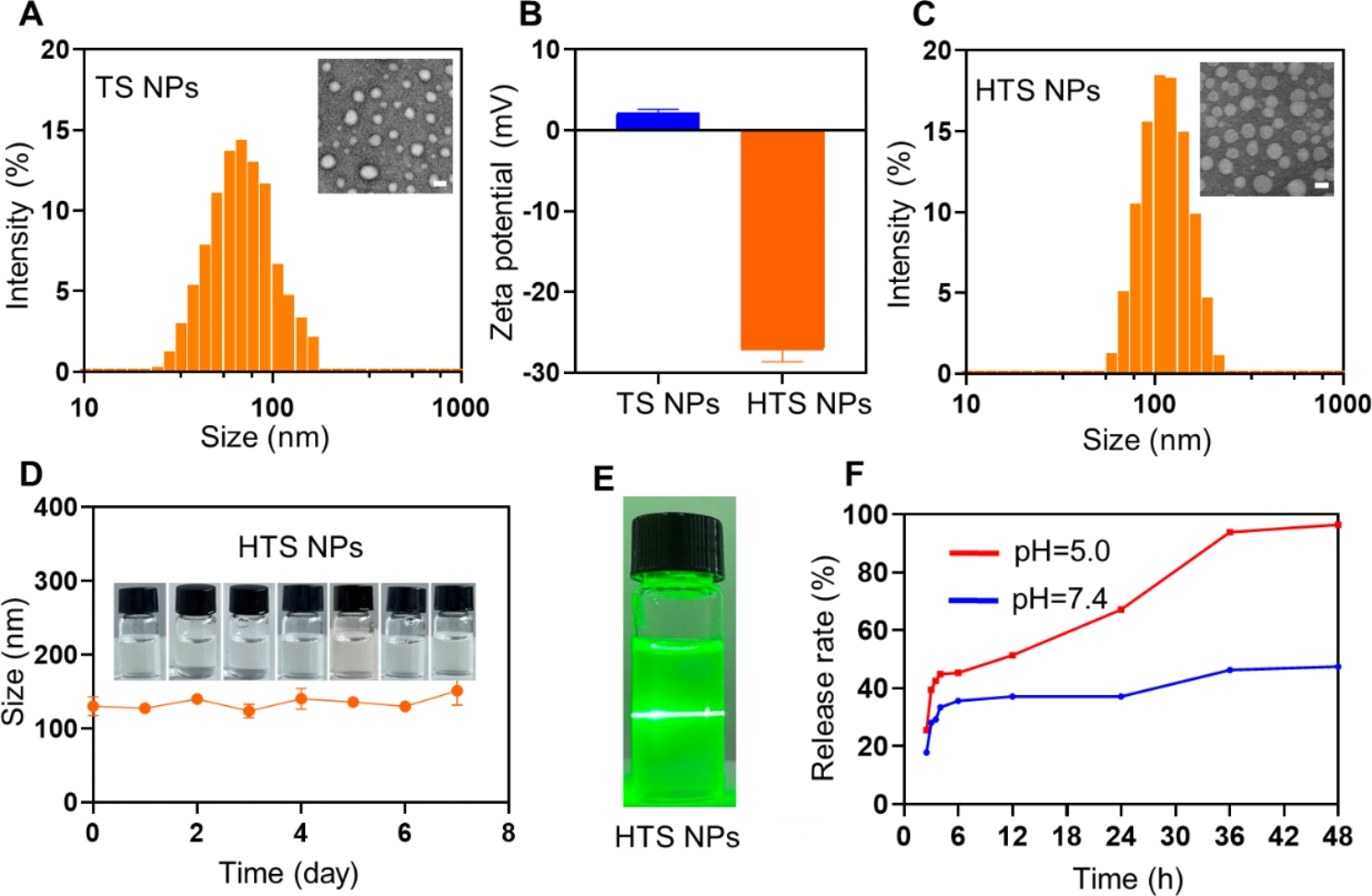
The characteristics of HTS NPs. (**A**) Size distribution of TS NPs (inset: TEM image of TS NPs, scale bar: 50 nm). (**B**) The zeta potential of TS NPs and HTS NPs. (**C**) Size distribution of HTS NPs (inset: TEM image of HTS NPs, scale bar: 50 nm). (**D**) Stability of HTS NPs (inset: the bright field image of HTS NPs). (**E**) The tyndall effect of HTS NPs. (**F**) The release of sertaconazole. The error bars represent the means ± SD (n = 3)

Nanoparticles were prepared by thin-film hydration as described in Scheme 1A. Both dynamic light scattering and transmission electron microscopy (TEM) (Fig. 1A) images confirmed that the TS NPs have spherical nanostructures with appropriate nano-sizes. We then measured the Zeta potential of the TS NPs and found it to be positively charged and there was a marked change in particle size within 7 days (Fig. S1A). Surface charge plays an important role in the in vivo transport of nanoparticles, which can affect their efficacy. Therefore, we modified its surface with HA to obtain a negatively charged HTS NPs (Fig. 1B), which could prolong the blood circulation time by reducing interactions with plasma proteins[25, 26]. The particle size of HTS NPs measured by DLS was 122.0 nm (Fig. 1C). The nanoparticles were observed by electron microscopy and found to be homogeneous, with a dispersed spherical shape in solution (Fig. 1C). The UV-vis absorbance spectra of HA, TPGS, STZ and HTS NPs were conducted to confirm the successful synthesis (Fig. S1B). Furthermore, we found that HTS NPs significantly improved the dispersion of STZ in aqueous solution, which is expected to improve bioavailability of the drug (Fig. S1C). Subsequently, we determined their stability and found that there was no significant difference in the particle size of the HTS NPs after 7 days of standing (Fig. 1D). In addition, the bright field image of HTS NPs dispersed in PBS also exhibited remarkable dispersibility and stability for 7 days (Fig. 1D) The Tyndall effect of HTS NPs was clearly visible (Fig. 1E), even 7 days later (Fig. S1D). Next, to investigate the release rate, standard curves for sertaconazole at different pH concentrations were constructed (Fig. S1E). The release of sertaconazole in HTS NPs was 38.28% and 98.38% at pH 7.4 and pH 5.0, respectively (Fig. 1F). The above results indicated that the HTS NPs had been successfully prepared and possessed excellent physicochemical properties.

### The cellular uptake and cytotoxicity of HTS NPs

**Fig. 2:**
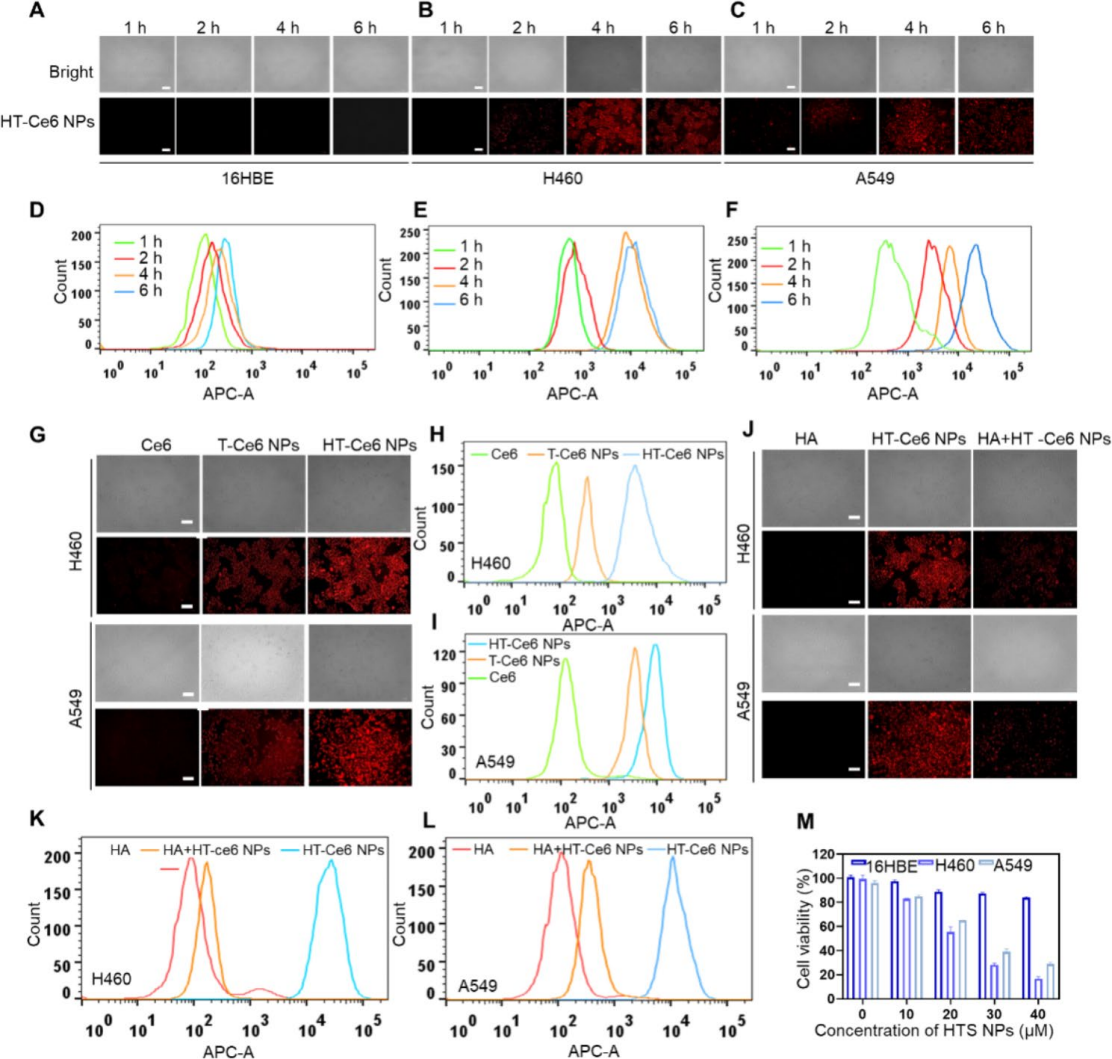
The cellular uptake and cytotoxicity of HTS NPs in vitro. (**A-C**) Confocal fluorescence images displaying cellular uptake of HTS NPs. Scale bar: 100 μm. (**D-E**) Flow cytometry analysis of the cellular uptake of HTS NPs. (**G-I**) Confocal fluorescence images and flow cytometry analysis showing cellular uptake of free Ce6, T-Ce6 NPs and HT-Ce6 NPs. Scale bar: 100 μm. (**J-L**) Confocal fluorescence images and flow cytometry analysis showing cellular uptake of HT-Ce6 NPs with or without HA. Scale bar: 100 μm. (**M**) The viability of 16HBE, H460 and A549 cells following treatment with HTS NPs. The error bars represent the means ± SD (n = 3)

The efficient accumulation of nanoplatforms in cells is a prerequisite for successful tumor suppression. Since HTS NPs do not have native fluorescence, we co-loaded Ce6 (a second-generation photosensitizer) to prepare HT-Ce6 NPs that could be detected by fluorescence. Confocal laser scanning microscopy (CLSM) and flow cytometry (FC) were used to evaluate the cell uptake time and tumor-related targeting ability of the HT-Ce6 NPs using the bronchial epithelial cell lines 16HBE and H460 and A549 lung cancer cells. As shown in Fig. 2A-F, CLSM analysis and corresponding FC analysis showed that HTS NPs had a time-dependent absorption behavior, reaching the strongest fluorescence intensity after 4 h, indicating efficient cell absorption after 4 h of treatment. The remaining cell-based experiments were treated with drug for 4 h. It is interesting to note that normal 16HBE lung epithelial had virtually no nanoparticle update.

Next, we compared the cellular uptake behavior of different preparations (Fig. 2G-I). Compared with free Ce6, the fluorescence intensity of T-Ce6 NPs and HT-Ce6 NPs in lung cancer cells was stronger, indicating that the tumor-related targeting ability was significantly improved after sertaconazole was incorporated into the nanoparticles. In addition, the uptake of nanoparticles by the pretreated cells was significantly reduced, indicating that the nanoparticles were targeted to tumor cells by binding the surface modified HA to the CD44 of tumor cells (Fig. 2J-L). These results clearly demonstrated that HTS NPs could serve as an ideal nanoplatform for efficient delivery of therapeutic agents to lung cancer cells. Preliminary in vitro studies were made to evaluate the anti-tumor effects of the nanoparticles against 16HBE, H460 and A549 cells. After treatment with various agents at different concentrations, the viability of both lung cancer cells decreased in a dose-dependent manner. Specifically, the cell viability of HTS NPs (40 µM) reduced to 20%. By contrast, HTS NPs showed little cytotoxicity in 16HBE cells (Fig. 2M).

### HTS NPs inhibit lung cancer cells growth in vitro

**Fig. 3:**
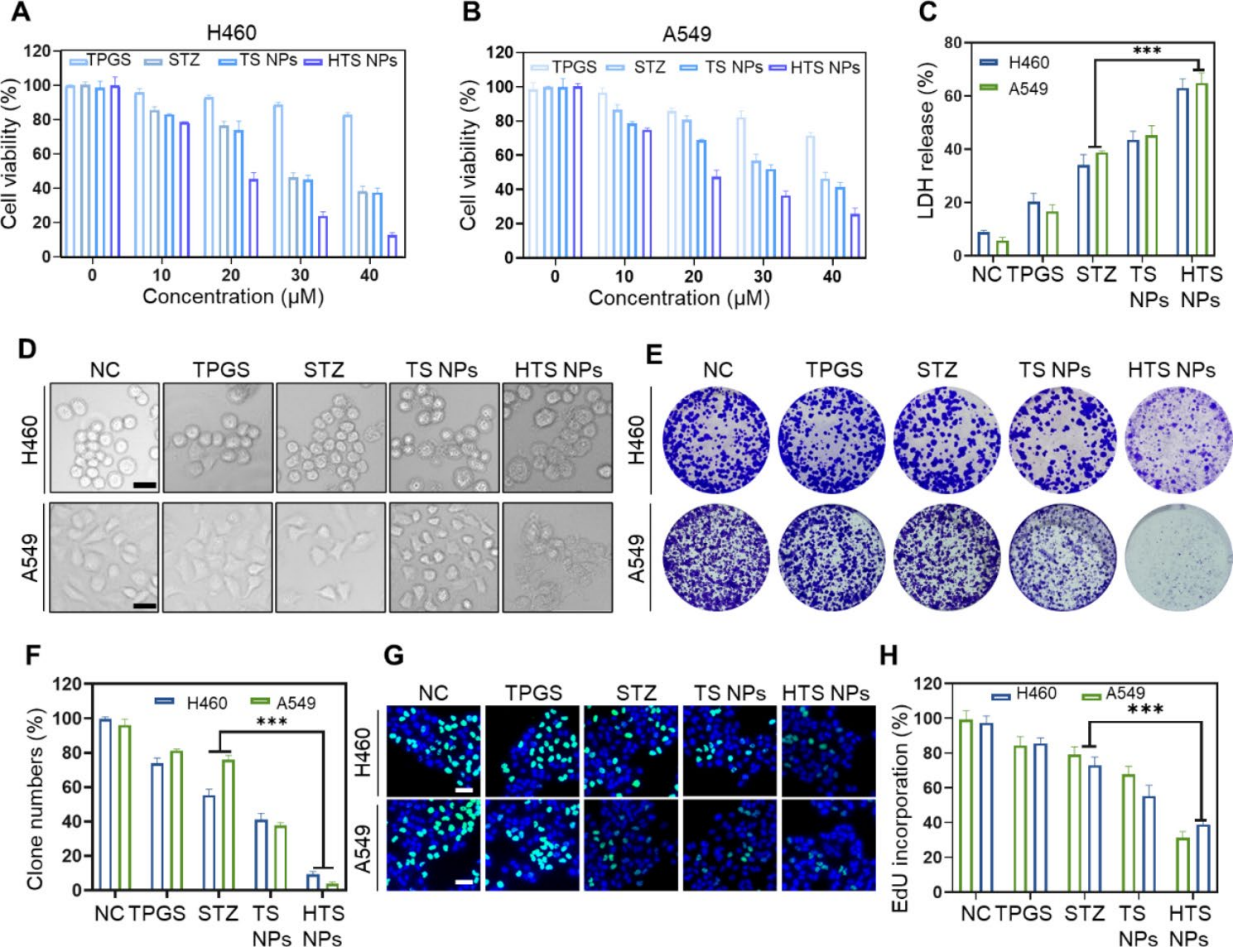
The anti-lung cancer effect of HTS NPs in vitro. (**A-B**) The viability of H460 and A549 cells following treatment with TPGS, STZ TS NPs and HTS NPs at different concentrations (n = 3). (**C**) LDH assay of H460 and A549 cells co-cultured with TPGS, STZ, TS NPs and HTS NPs (n = 3). (**D**) Images of the effects of morphology on H460 and A549 lung cancer cells with different formulations. Scale bar: 50 μm. (**E-F**) Colony formation assay and quantitative analysis of H460 and A549 lung cancer cells (n = 3). (**G-H**) H460 and A549 cells co-cultured with different groups using the EdU assay (n = 3). Scale bar: 50 μm. The error bars represent the means ± SD (***P < 0.001)

Subsequently, the toxic effect of HTS NPs on lung cancer cells was further verified. The anticancer activity of HTS NPs in different treatment groups (including STZ TPGS, TS NPs and HTS NPs) was determined using the 3-(4,5- dimethylthiazole - 2- yl) -2,5- diphenyl tetrazole bromide method [27]. The results showed that cell activity decreased with increasing HTS NPs concentration, and the HTS NPs significantly inhibited the growth of H460 and A549 lung cancer cells compared to sertaconazole monotherapy (Fig. 3A-B). Similarly, LDH release experiments showed significant cytotoxicity of HTS NPs in lung cancer cells (Fig. 3C). Subsequently, morphological changes in the lung cancer cells were observed after treatment with different dosage groups of sertaconazole as the carrier, TPGS, TS NPs and HTS NPs (Fig. 3D). In addition, the proliferation of lung cancer cells was significantly inhibited after treatment with HTS NPs. The results of the cloning experiments showed that HTS NPs had the weakest cloning ability under the treatment of different preparation groups (Fig. 3E-F). Similarly, EdU experimental results also showed that HTS NPs could significantly reduce the positive rate of EdU labeling for lung cancer cells (Fig. 3G-H). These data indicated that the HTS NPs show significant growth inhibition of lung cancer cells in vitro.

### HTS NPs-mediated ROS induces cytotoxic autophagy in lung cancer cells

**Fig. 4:**
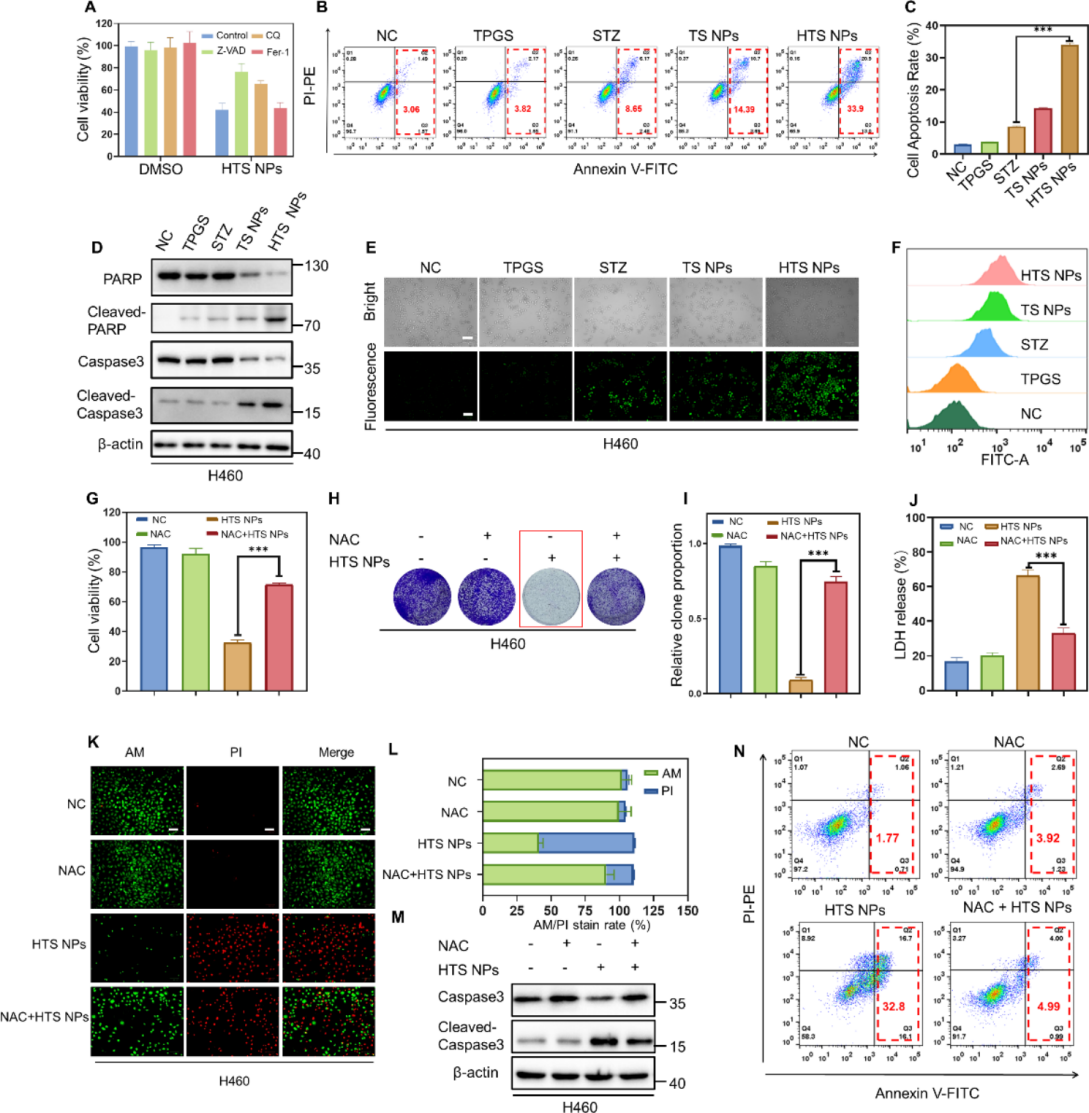
HTS NPs-mediated ROS induce lung cancer cell death. (**A**) MTT assay of H460 cells treated with the HTS NPs and in combination with or without Z-VAD, Fer-1 and CQ (n = 3). (**B-C**) Annexin V-FITC/PI staining analysis of apoptosis by flow cytometry (n = 3). (**D**) Western blot analysis of apoptotic markers for H460 cells treated with TPGS, STZ, TS NPs and HTS NPs. (**E-F**) ROS generation in H460 cells after different treatments using fluorescence imaging and flow cytometry. Scale bar: 100 μm. (**G**) MTT assay, colony formation assay (**H-I**) and LDH assay (**J**) of H460 cells were treated with HTS NPs with or without NAC (2 µM) treatment (n = 3). (**K-L**) Fluorescence imaging of AM/PI staining assay and quantification analysis (**L**) in lung cancer cells after different treatments with HTS NPs with or without NAC (2 µM), scale bar: 100 μm. (**M**) Immunoblot analysis of apoptotic markers for H460 cells treated HTS NPs with or without NAC treatment. (**N**) Annexin V-FITC/PI staining analysis of apoptosis H460 cells by flow cytometry. The error bars represent the means ± SD (n = 3, ***P < 0.001)

To determine the molecular mechanism of HTS NPs-induced lung cancer cell growth inhibition, several inhibitors with different cell death patterns were used to evaluate their role in nanoparticle-induced tumor suppression. It was found that Ferrostatin-1 (Fer-1) (an effective and selective inhibitor of ferroptosis) had no significant effect on cell growth in the presence of HTS NPs, while the combined use of Z-VAD (an inhibitor of apoptosis), or chloroquine (CQ, an autophagy inhibitor), with nanoparticles alleviated the inhibition of lung cancer cell growth, particularly Z-VAD (Fig. 4A, S2A). To further assess whether the cytotoxic effect of HTS NPs was associated with apoptosis, flow cytometry analysis of the apoptotic rate using Annexin V/PI staining showed a marked increase in apoptosis induction in lung cancer cells after HTS NPs treatment (Fig. 4B-C, S2B). In addition, increased cleavage of caspase 3 and PARP was also observed in HTS NPs-treated cancer cells at the protein level (Fig. 4D, S2C). The above results suggested that HTS NPs play an anti-tumor role in lung cancer cells by inducing apoptosis.

In addition, as shown in Fig. 4A and S2A, we found that an autophagy inhibitor (CQ) also reversed the inhibition of lung cancer cells induced by HTS NPs, suggesting that autophagy may be stimulated, thus triggering pro-apoptotic autophagy in lung cancer cells. Therefore, we studied whether HTS NPs can induce autophagy. One of the signs of autophagy is the conjugation of LC3B-I with PE and conversion to LC3B-II, which is necessary for the induction of autophagy. Next, we detected the protein expression levels of autophagy-related genes in lung cancer cells treated with different preparations. As shown in Fig. S3A-B, the level of LC3B-II in H460 and A549 cells was increased after HTS NPs treatment compared with that in the free sertaconazole group. However, the protein levels of p62 and BCL-2 decreased, which implied the induction of complete autophagic flux. Notably, treatment with 3-MA or CQ also alleviated HTS NPs-induced apoptosis in H460 and A549 cells (Fig. S3C), suggesting that HTS NPs induced pro-apoptotic autophagy after treatment. Taken together, these results suggest that HTS NPs-induced apoptosis and pro-apoptotic autophagy contribute to its anticancer effects.

Evidence suggests that intracellular ROS accumulation can induce cancer cell apoptosis [28, 29]. To further explore the mechanism of HTS NPs-induced apoptosis, we subsequently evaluated intracellular ROS levels by fluorescence imaging and flow cytometry using the DCFH-DA probe. The results showed a significant increase in intracellular ROS in tumor cells treated with HTS NPs (Fig. 4E-F, S2D-E). To determine the role of ROS accumulation in HTS NPs-induced apoptosis, we treated cells with the ROS scavenger n-acetylcysteine (NAC) and found that it significantly restored nanoparticle-induced growth inhibition in the form of increased cell viability and clonal formation (Fig. 4G-I, S2F-H). LDH results also indicated that NAC eliminated the toxic effects of HTS NPs on lung cancer cells (Fig. 4J, S2I). As shown in Fig. 4K-L and S3J-K, the live/dead staining assays also supported that HTS NPs-mediated ROS induction contributing to cancer cell death. Notably, NAC combined with nanoparticle treatment enhanced the expression of oxidative stress-related proteins Nrf-2, HO-1 and SOD1 in lung cancer cells (Fig. S4A). Intracellular T-AOC is regulated by external stimuli and reflects the antioxidant capacity of drugs. In lung cancer cells treated with HTS NPs, the activity of T-AOC was significantly inhibited compared with the control, which could be restored after co-incubation of cells with NAC (Fig. S4B). Similarly, the inhibition of Superoxide Dismutase (SOD), an important antioxidant enzyme in the body, was significantly reversed by HTS NPs combined with NAC (Fig. S4C). In addition, we found that NAC could inhibit the increase of oxidative product MDA induced by HTS NPs (Fig. S4D). In addition, NAC treatment also reduced the pro-apoptotic effect of HTS NPs (Fig. 4M-N, S2L-M). In summary, these results indicate that HTS NPs promote apoptosis of lung cancer cells by inducing excessive accumulation of intracellular ROS.

### In vivo anti-tumor effects of HTS NPs

Good compatibility with red blood cells is a necessary condition for the in vivo application of biomaterials [30]. Therefore, the compatibility of sertaconazole and different concentrations of HTS NPs with mouse red blood cells was studied using a hemolysis test. The erythrocytes of the NPs-treated mice were less damaged, and the hemolysis rates were less than 5% in all groups. The hemolysis rate of nanoparticles at the same concentration was lower than that of the single drug (the hemolysis rate was about 8% after the single drug treatment) (Fig. S5A-B). It indicated that NPs had favorable compatibility with mouse red blood cells.

**Fig. 5:**
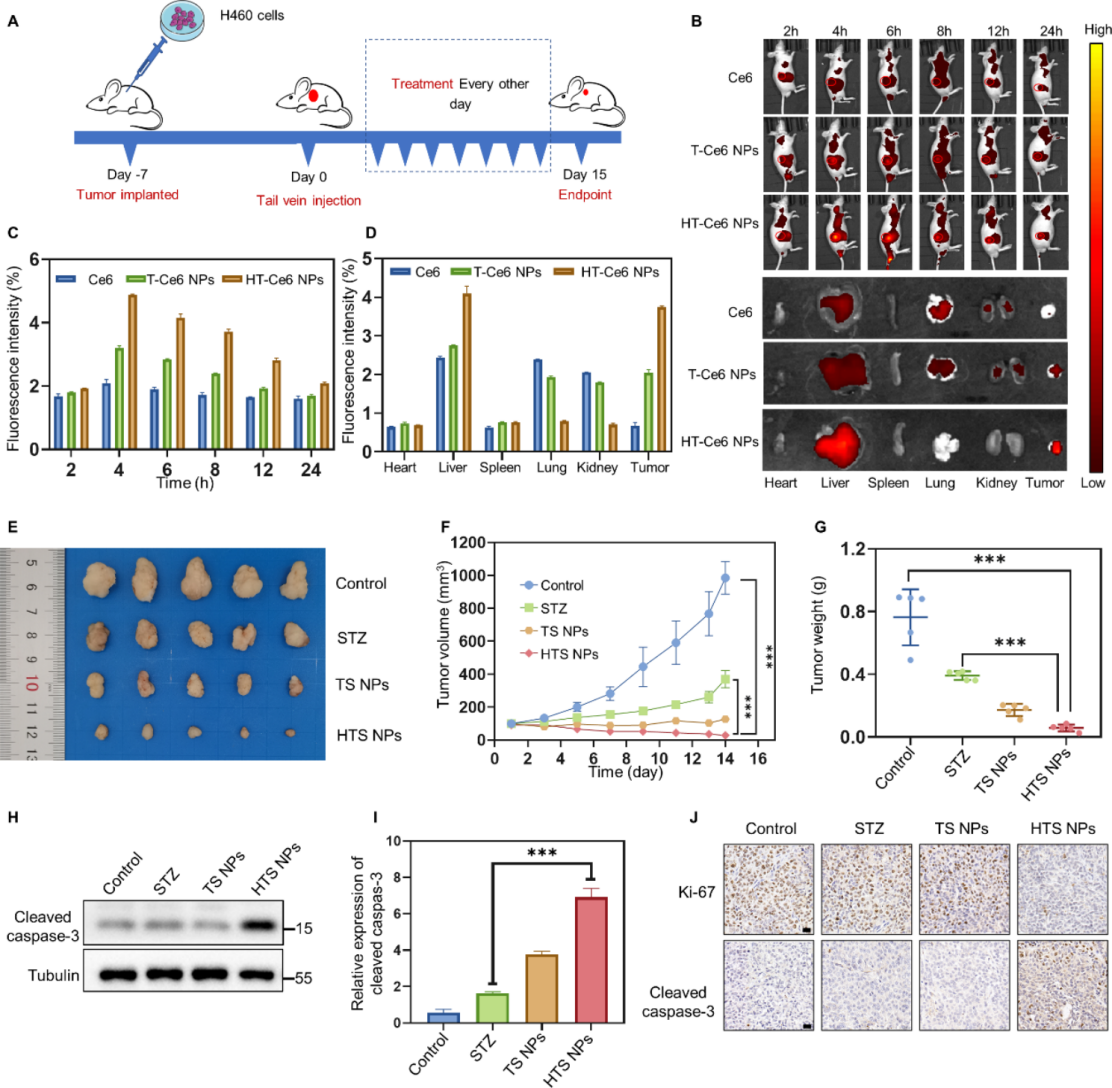
The anti-lung cancer effect of HTS NPs in vivo. (**A**) Schematic illustration of the establishment and treatment regime of the H460 tumor bearing BALB/c nude mice model. (**B**) In vivo biodistribution of Ce6, T-Ce6 NPs and HT-Ce6 NPs at indicated time points. (**C-D**) The fluorescence intensity was calculated and analyzed based on the data in (**B**). (**E**) Representative images of isolated tumors (n = 5). (**F**) Tumor volume curves of different groups (n = 5). (**G**) The tumor weight of the different groups (n = 5). (**H-I**) Western blot analysis of the cleaved-caspase 3 levels in these tumor tissues obtained from different groups at the 14th day. (**J**) Ki-67 and cleaved-caspase 3 immunohistochemistry images of mice treated with various treatment groups (Scale bars: 20 μm). The error bars represent the means ± SD (***P < 0.001)

To test the therapeutic effect of HTS NPs in vivo, we established the H460 cell subcutaneous tumor model, and then randomly divided H460 tumor-bearing mice into four groups, which were injected with PBS, STZ TS NPs and HTS NPs, respectively, to evaluate the anti-tumor effects of different treatment regimens (Fig. 5A). A fluorescence living imaging system was applied to monitor the fluorescence signal of Ce6, T-Ce6 NPs and HT-Ce6 NPs after intravenous tail injection into H460 tumor-bearing mice at different time points. Fluorescence imaging showed that HT-Ce6 NPs exhibited an excellent tumor accumulation ability, and a stronger fluorescence intensity in the tumor sites compared with T-Ce6 NPs and Ce6 group (Fig. 5B-D). These results indicated that HT-Ce6 NPs could specifically target the tumor sites, resulting in increases in the drug aggregation at the tumor sites. Tumor size and tumor volume were recorded 14 days after treatment. The mice were sacrificed 14 days after treatment and the tumors were removed for weighing and photographing. Results for tumor volume and tumor weight showed varying degrees of tumor inhibition, with HTS NPs being the most effective (Fig. 5E-G). The tumor protein is separate for the next experiment. Noteworthy, HTS NPs treatment enhanced the expression of the apoptosis marker cleaved-caspase 3 in tumor tissues, which was consistent with the results in vitro, indicating that it also induced apoptosis in vivo to inhibit tumor growth (Fig. 5H-I). In addition, IHC staining of Ki-67 and cleaved-caspase 3 in tumor tissue further confirmed the anti-proliferative and pro-apoptotic properties of HTS NPs (Fig. 5J). These data suggest that our nanoparticles inhibit tumor growth in vivo by inducing apoptosis in tumor tissues.

### HTS NPs increased ROS levels in vivo

**Fig. 6:**
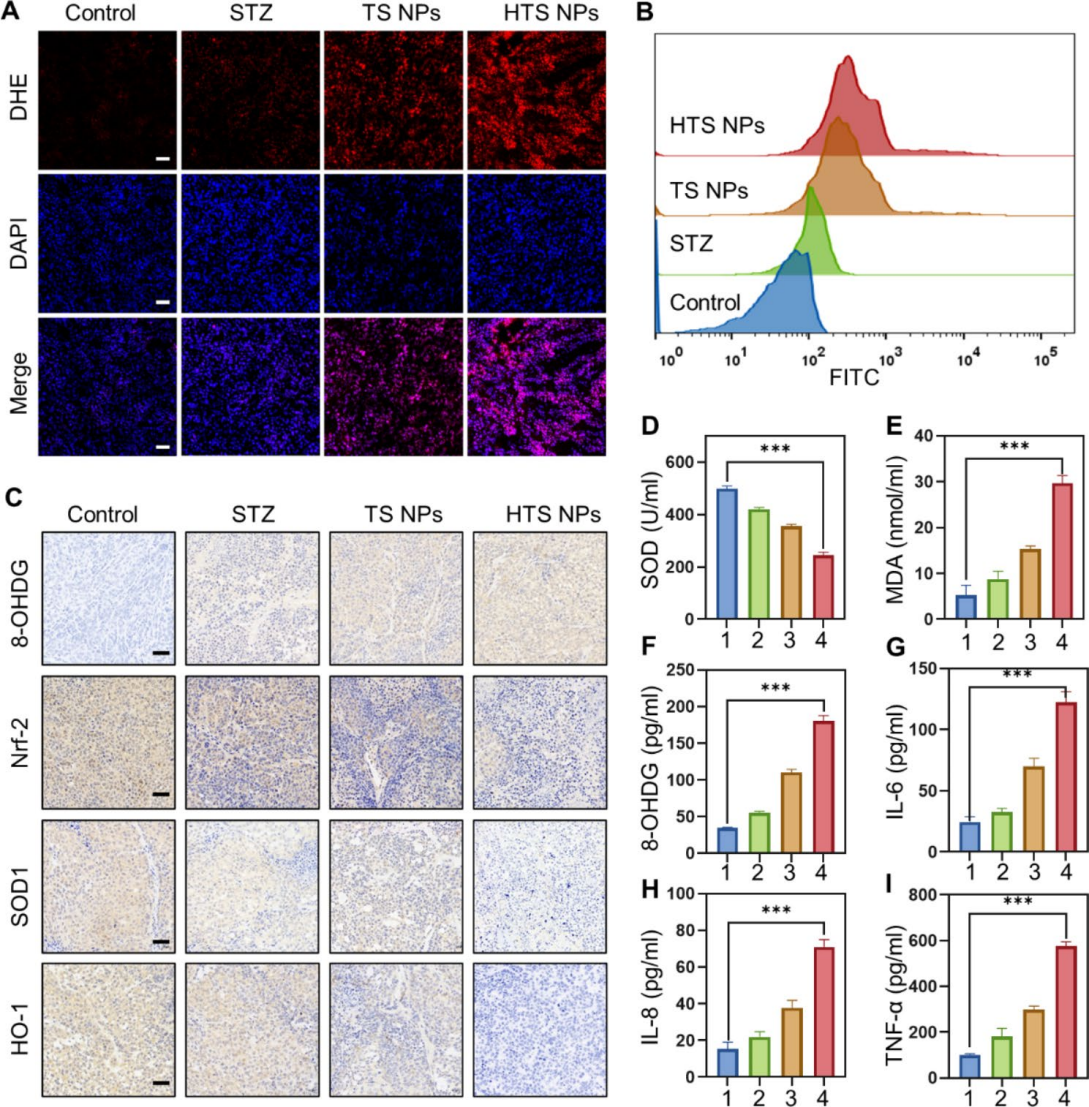
The levels of ROS in vivo. (**A**) Staining of tumor tissue sections harvested from different treated mice with ROS probe DHE (red) (scale bar: 50 μm) and flow cytometry assay (**B**). (**C**) IHC stain of 8-OHdG, Nrf-2, SOD1 and HO-1 of tumor tissues; scale bar: 50 μm (n = 5). (**D-J**) Elisa assay of MDA (**D**), SOD (**E**),8-OHDG (**F**) and IL-6 (**G**), IL-8 (**H**), and TNF-α (**I**) in mice serum (n = 3). 1:Control; 2:STZ; 3:TS NPs; 4:HTS NPs. The error bars represent the means ± SD (***P < 0.001)

To further verify the involvement of ROS in the process of HTS NPs-induced cancer cell death in vivo. Frozen section analysis of tumor samples revealed a significant increase in DHE staining, indicating elevated ROS levels in mice treated with HTS NPs (Fig. 6A-B). IHC staining of 8-OHDG, Nrf-2, SOD1 and HO-1 showed the ability of HTS NPs to induce oxidative damage in tumor tissues (Fig. 6C). Furthermore, elisa results showed that MDA and 8-OHDG were significantly increased, while the antioxidant enzyme SOD was decreased (Fig. 6D-F). In addition, the positive release of inflammatory factors such as IL-6, IL-8 and TNF-α in the serum of tumor-bearing mice confirmed the contribution made by HTS NPs to induce oxidative stress in vivo (Fig. 6G-I). The above results suggest that ROS is also involved in the treatment of HTS NPs in vivo. Moreover, these results strongly supported that ROS played a significant role in HTS NPs mediated antitumor efficacy in vivo.

### The evaluation of biosafety of HTS NPs in vivo

**Fig. 7:**
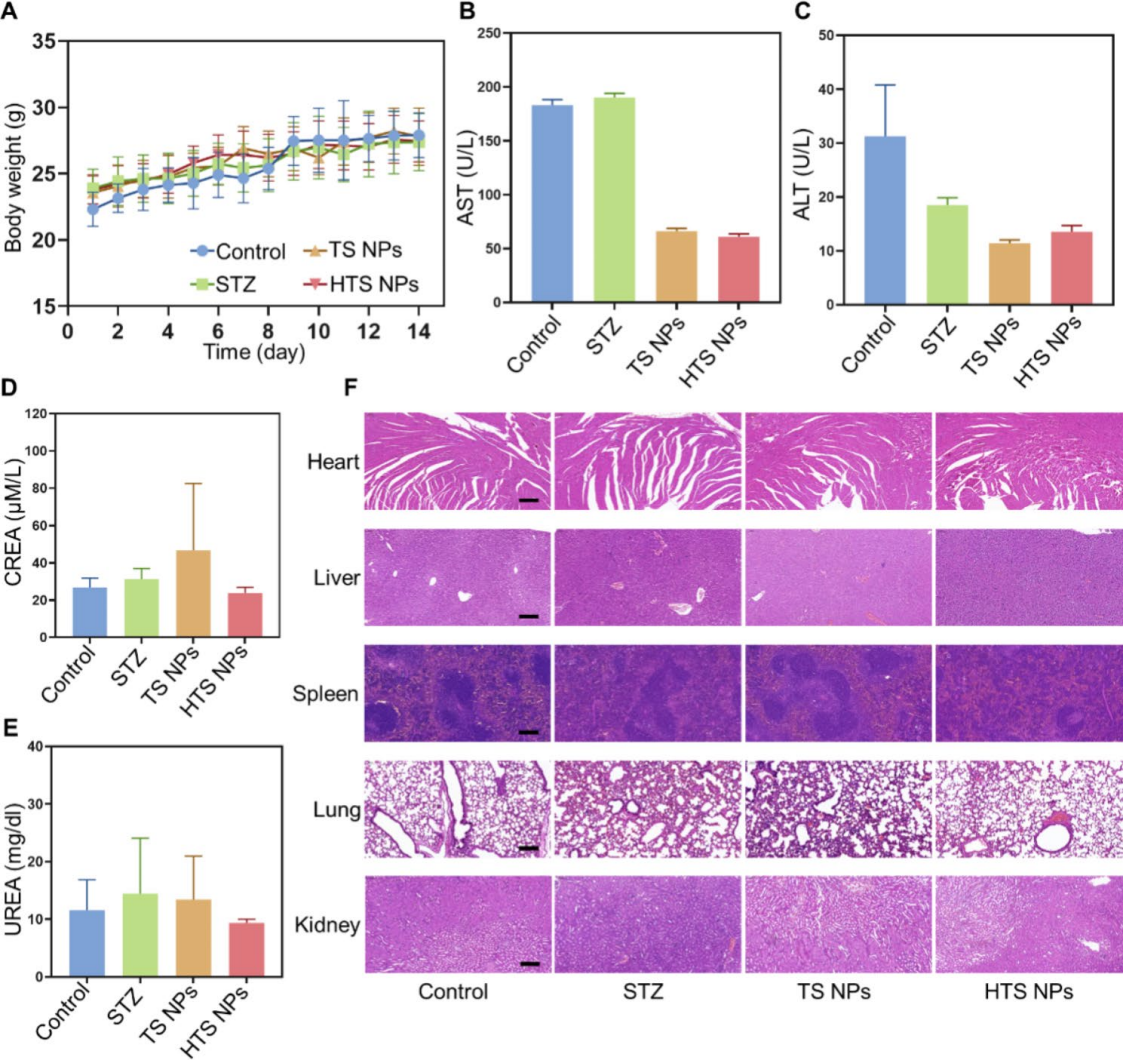
The biocompatibility of HTS NPs in vivo. (**A**) Body weight of different groups of tumor-bearing BALB/c nude mice (n = 5). (B-E) The serum biochemistry analysis (AST (**B**); ALT (**C**); CREA (**D**); UREA (**E**)) of mice with different treatments (n = 3). (**F**) The H&E staining of the major organs in mice after different treatments (Scale bars = 50 μm). All data are shown as mean ± SD and are representative of 3 independent experiments. The error bars represent the means ± SD (n = 5)

The body weight fluctuation of the mice in the different treatment groups was negligible, indicating that our nanoparticles were biosafe (Fig. 7A). In addition, alanine aminotransferase and aspartate aminotransferase (ALT/AST) are commonly used blood biomarkers reflecting liver function, while blood creatinine and urea nitrogen (CREA/UREA) are widely used to monitor renal function [31, 32]. In our study,

we found that these indexes were within their normal range [33] and had no significant fluctuation among all groups, suggesting low side-effect of the HTS NPs (Fig. 7B-E). Next, we performed histopathological analysis of heart, liver, spleen, lung and kidney by using hematoxylin-eosin (H&E) staining. The results showed no pathological damage to major organs after HTS NPs treatment (Fig. 7F). All these results already indicated that HTS NPs had decent biosecurity properties.

## Conclusions

In summary, we repurposed the antifungal drug sertaconazole as a new nanomedicine agent for the treatment of lung cancer. The designed HTS could improve the water solubility of sertaconazole and enhance its anti-tumor effect, which increases the potential of clinical translation. Specifically, the HTS NPs acts as a pH-responsive drug delivery platform to promote the intracellular release of TPGS and sertaconazole and induce apoptosis by triggering excessive ROS accumulation to inhibit the growth of tumor cells. Our experimental results on anti-cancer activity in vitro and in vivo have shown that HTS NPs could significantly inhibit the proliferation of tumor cells. Importantly, HTS NPs are biologically safe for other major organs. Therefore, the nanoplatform has the potential to become a new strategy to improve therapeutic effects. In addition, this research has shown that repurposing old drugs with nanomedicine technologies could be an attractive method in the discovery of anti-tumor drugs.

## Electronic supplementary material

Below is the link to the electronic supplementary material.

Supplementary material 1

## Data Availability

All the data obtained and/or analyzed during the current study were available from the corresponding authors on reasonable request.
