# Supplementary material for: Sertaconazole-repurposed nanoplatform enhances lung cancer therapy via CD44-targeted drug delivery
Source: J Exp Clin Cancer Res. 2023 Jul 29;42:188. doi: 10.1186/s13046-023-02766-2 (PMC10385912; doi:10.1186/s13046-023-02766-2)
Supplement: Supplementary file 1 — Supplementary material 1 [file 13046_2023_2766_MOESM1_ESM.docx]

**Supporting information**

**
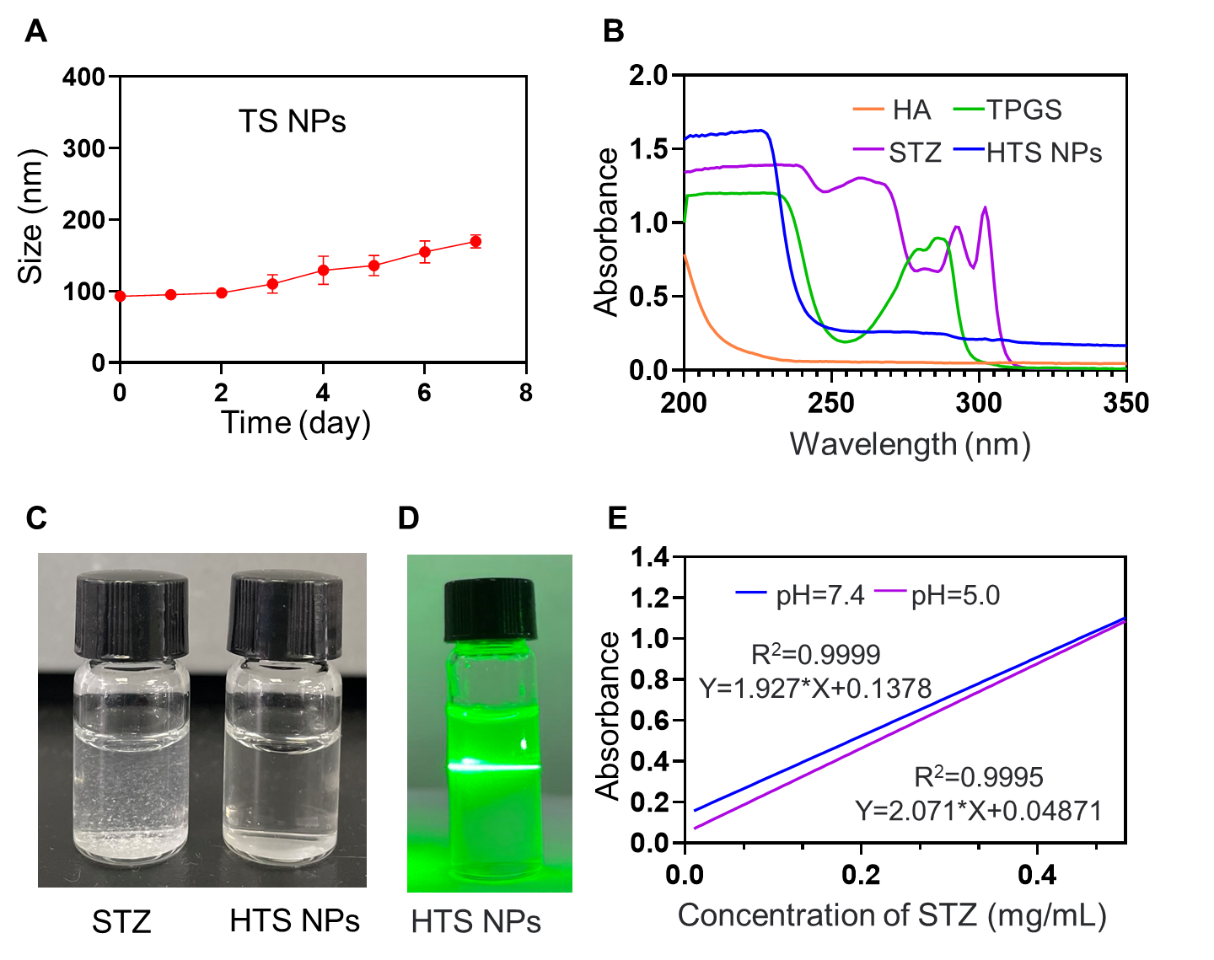
**

**Fig. S1.** **Synthesis and characterization of HTS NPs.** (**A**) Stability of TS NPs. (**B**) UV–vis absorption spectra of HA, TPGS, STZ and HTS NPs. (**C**) Sertaconazole suspension (left) and HTS NPs (right). (**D**) The tyndall effect of HTS NPs after 7 days at room temperature. (**E**)The standard curve of STZ at pH 5.0 and pH 7.4. The error bars represent the means ± SD (n = 3).


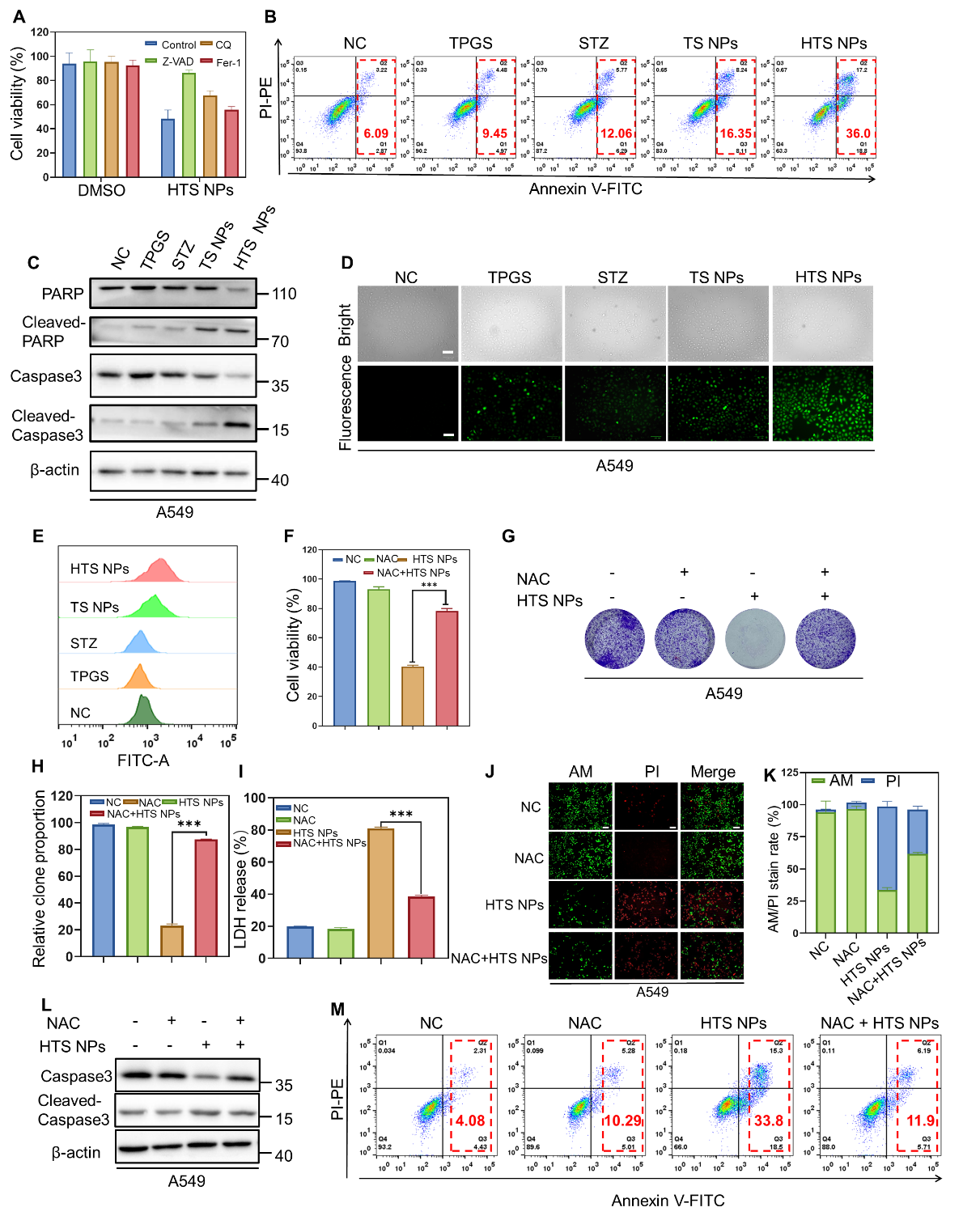


**Fig. S2. HTS NPs induces excessive ROS accumulation to cause apoptosis in A549.** (**A**) MTT assay of A549 cells treated with the HTS NPs and in combination with or without Z-VAD, Fer-1 and CQ. (**B**) Annexin V-FITC/PI staining analysis of apoptosis by flow cytometry. (**C**) Western blot analysis of apoptotic markers for A549 cells treated with TPGS, STZ, TS NPs and HTS NPs. (**D-E**) ROS generation in A549 cells after different treatments using fluorescence imaging and flow cytometry. (**F**) MTT assay, colony formation assay (**G-H**) and LDH assay (**I**) of A549 cells were treated with HTS NPs with or without NAC (2 μM) treatment (n = 3). (**J-K**) Fluorescence imaging of AM/PI staining assay and quantification analysis (K) in lung cancer cells after different treatments with HTS NPs with or without NAC (2 μM), scale bar: 100 µm. (**L**) Immunoblot analysis of apoptotic markers for A549 cells treated HTS NPs with or without NAC treatment. (**M**) Annexin V-FITC/PI staining analysis of apoptosis A549 cells by flow cytometry. The error bars represent the means ± SD (n = 3, ****P* < 0.001).


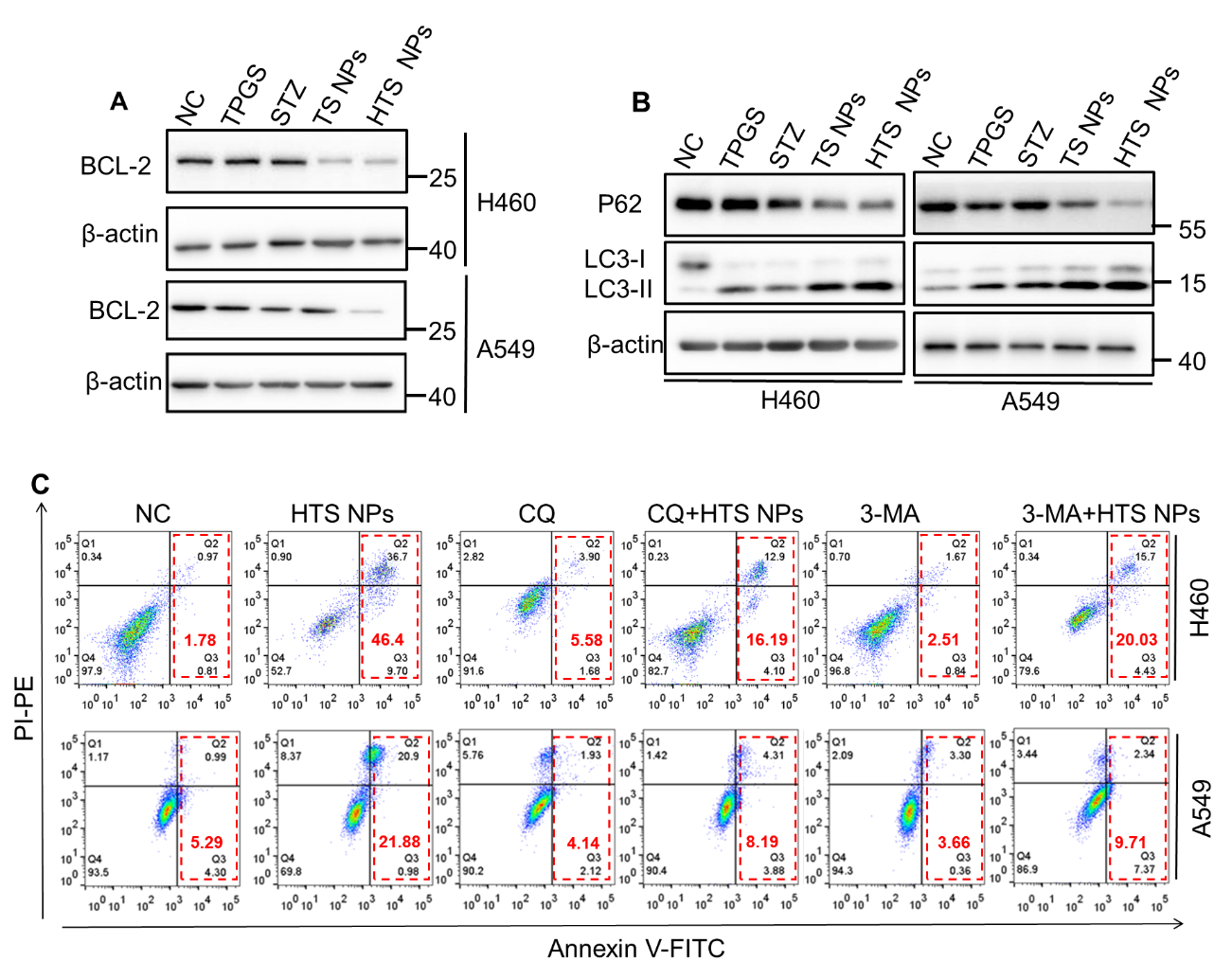


**Fig. S**3**. HTS NPs induces autophagy in NSCLC cells.** (**A**) Immunoblotting of, p62 and LC3 turnover in A549 and H460 cells treated with TPGS, free STZ, TS NPs and HTS NPs. (**B**) A549 and H460 cells were treated with HTS NPs in the absence or presence of CQ or 3-MA, and (**C**) flow cytometry was performed to evaluate the apoptotic cells.


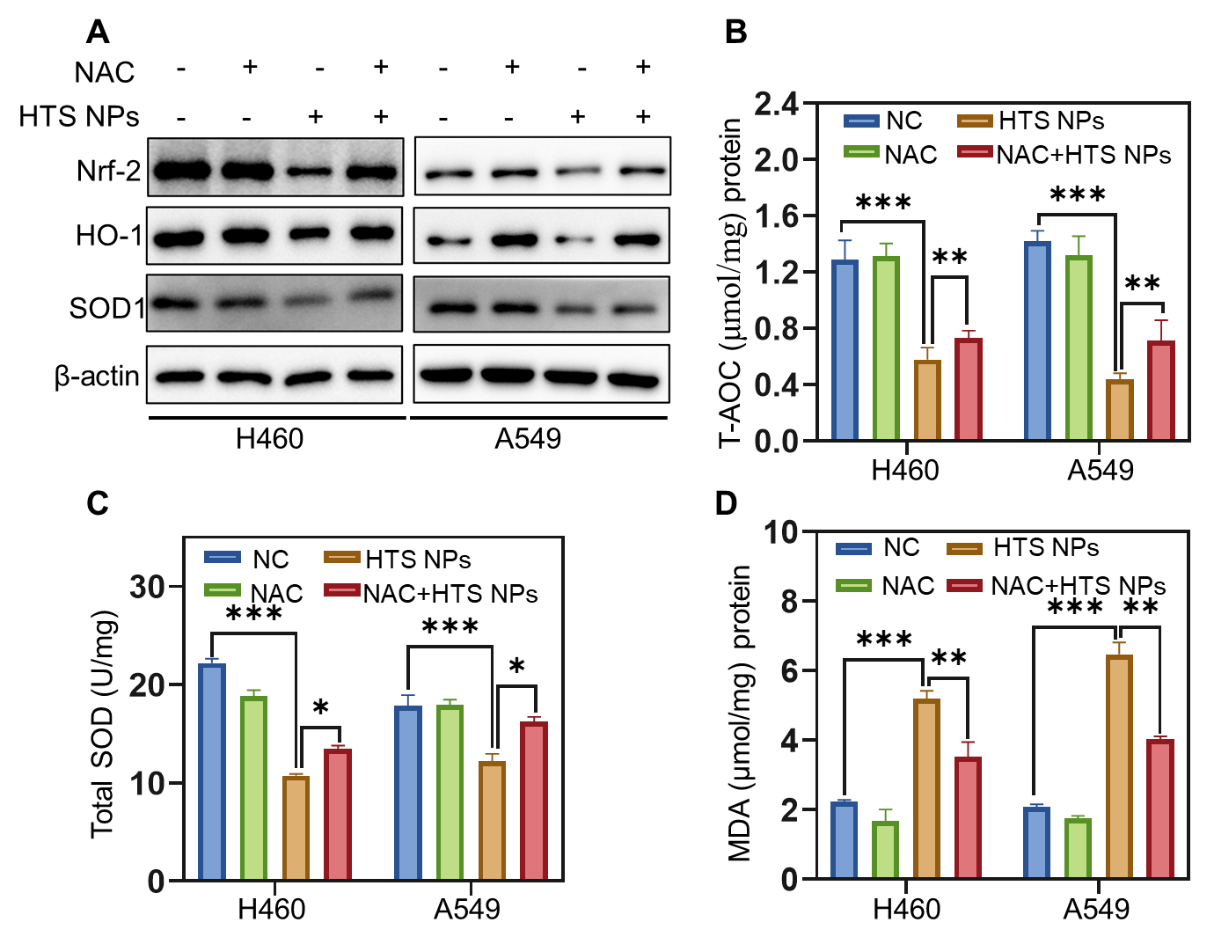


**Fig. S4.** **HTS NPs** **induce lung cancer cell death via ROS.** (**A**) Immunoblot analysis of Nrf-2, HO-1 and SOD1 levels. (**B**) Cellular T-AOC quantification. (**C**) Analysis of total SOD. (**D**) Analysis of MDA generation. The error bars represent the means ± SD (n = 3, **P* < 0.05, ***P* < 0.01, ****P* < 0.001).


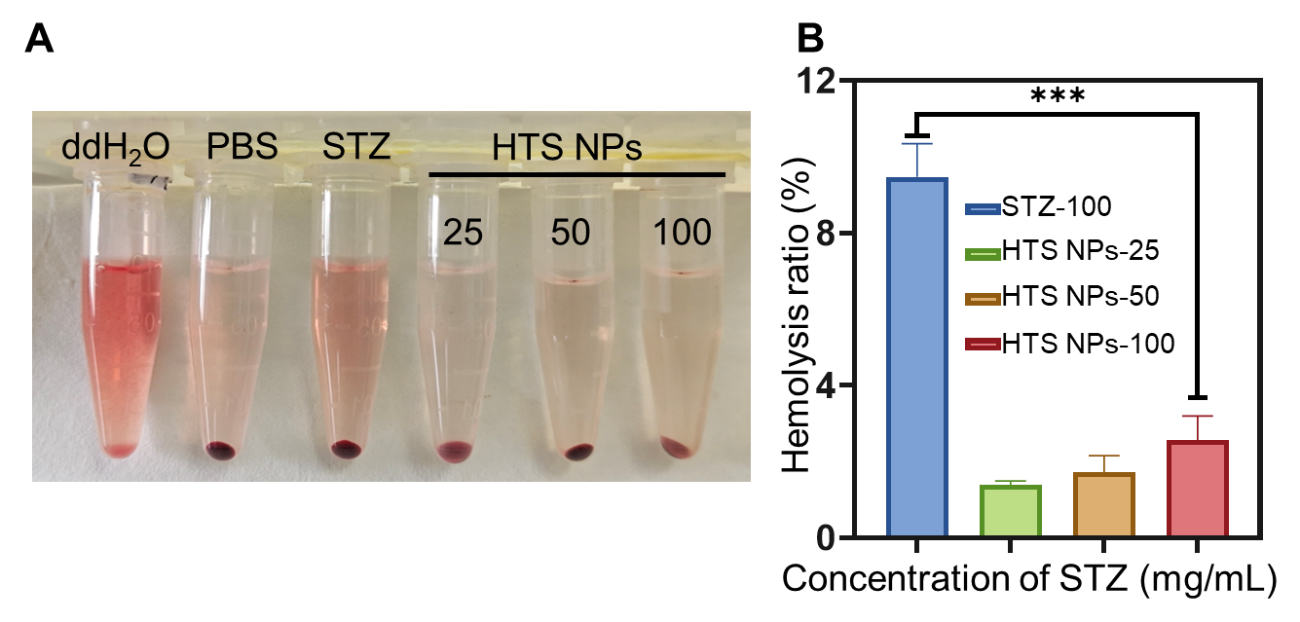


**Fig. S5.** **The hemolysis test. (A**) The images of red blood cells after incubation with different concentrations for 2 h and hemolysis rates (**B**) of RBCs from mice treated with ultrapure water (ddH_2_O) and PBS (pH 7.4) groups were used as control (n = 3). The error bars represent the means ± SD (****P* < 0.001).
